# Establishment of an Early Prediction Model for Severe Fever With Thrombocytopenia Syndrome‐Associated Encephalitis

**DOI:** 10.1002/iid3.70096

**Published:** 2024-12-11

**Authors:** Yijiang Liu, Naisheng Zhu, Zimeng Qin, Chenzhe He, Jiaqi Li, Hongbo Zhang, Ke Cao, Wenkui Yu

**Affiliations:** ^1^ Department of Critical Care Medicine Nanjing Drum Tower Hospital Clinical College of Nanjing Medical University Nanjing China; ^2^ Department of Emergency Medicine, Nanjing Drum Tower Hospital, Affiliated Hospital of Medical School Nanjing University Nanjing China; ^3^ Southwest Medical University Luzhou China; ^4^ Department of Critical Care Medicine, Affiliated Hospital of Medical School, Nanjing Drum Tower Hospital Nanjing University Nanjing China

**Keywords:** encephalopathy, risk factor, score model, severe fever with thrombocytopenia syndrome

## Abstract

**Background:**

Severe fever with thrombocytopenia syndrome (SFTS) is an emerging infectious disease primarily transmitted by ticks. The development of encephalitis in SFTS patients significantly increases the risk of adverse outcomes. However, the understanding of SFTS‐associated encephalitis (SFTSAE) is still limited. This study aimed to identify the clinical characteristics of SFTSAE and develop a predictive model for early detection.

**Methods:**

We retrospectively collected data from 220 SFTS patients admitted to Nanjing Drum Tower Hospital between May 2019 and January 2024. The patients were first randomly divided into a training set (154 people, 70%) and a validation set (66 people, 30%). The patients in the training set were divided into SFTSAE and non‐SFTSAE groups according to the presence of encephalitis. A prediction model was constructed using the training set: important clinical parameters were selected using univariate logistic regression, and then multivariate logistic regression was performed to determine the independent risk factors for SFTSAE. A prediction model was constructed using these independent risk factors. Finally, the validation set was used to verify the predictive ability of the model.

**Results:**

Age, C‐reactive protein, d‐dimer, and viral load were independent risk factors for SFTSAE (*p* < 0.05). A nomogram containing these four indicators was constructed, and the predictive performance of the nomogram was evaluated using the ROC curve. The AUC of the model was 0.846 (95% confidence interval [CI]: 0.770–0.921), which had good predictive ability for SFTSAE.

**Conclusion:**

Conclusion: The overall mortality rate of SFTS patients was 17.53%, and the mortality rate of encephalitis patients was 50%. Old age, high C‐reactive protein, elevated d‐dimer, and high viral load were independent risk factors for SFTSAE. The nomogram constructed based on these four indicators had good predictive ability and could be used as an evaluation tool for clinical treatment.

## 1 Introduction

Severe fever with thrombocytopenia syndrome (SFTS) is an emerging infectious disease caused by a novel Bunyavirus primarily transmitted by ticks. It was first discovered by Chinese researchers in 2011 [1], named SFTS virus by the International Committee on Taxonomy of Viruses in 2014, and later renamed Dabie bandavirus in 2019. The virus belongs to the family *Bunyaviridae*, genus *Phlebovirus*, species *Severe fever with thrombocytopenia syndrome virus* (SFTSV). Since its discovery, this disease has been reported in multiple countries [2, 3, 4]. Patients with SFTS can exhibit a variety of symptoms, including fever, muscle pain, and fatigue, while critically ill patients may even exhibit bleeding tendencies, multiorgan failure, and impaired consciousness leading to death [5, 6]. SFTS has a mortality rate of 16.2%–30% [7]. SFTS has gained international attention and was listed as one of the top ten priority infectious diseases by the World Health Organization in 2017 [8].

Inflammation plays a crucial role in the occurrence and development of SFTS, and previous studies have attempted to elucidate this pathological process [9, 10]. Excessive inflammatory responses can even serve as a distinguishing factor between SFTS and other diseases, further highlighting the unique role of inflammation in the occurrence and development of SFTS [11]. However, the impact of excessive inflammation on SFTS patients remains insufficiently studied. In our research, we identified a correlation between the inflammatory response and the nervous system in SFTS patients, which provides further insights into the role of inflammation in the pathological process of SFTS and underscores the importance of controlling inflammation in these patients. This will have implications for future clinical practice.

Patients with SFTS may present with various central nervous system symptoms such as headaches, confusion, seizures, or even coma [7]. The presence of central nervous system symptoms in SFTS patients has been identified as an independent risk factor for adverse outcomes in several studies [7, 12, 13]. According to current reports, the incidence of SFTS‐associated encephalitis (SFTSAE) is 19.1%–22.7%, with a mortality rate of 44.7%–45.4% among encephalitic patients [14, 15], which is significantly higher than that of patients without encephalitis. Since there are currently no effective targeted treatments or vaccines for SFTS, early detection of potential encephalitis patients may be crucial in the management of SFTS.

The nomogram is a widely used prediction model. It can integrate multiple predictive indicators and use line segments with scales to display them on the same image in a certain proportion to predict the model's outcome. For example, nomograms have been used in a variety of diseases such as cancer, heart failure, kidney failure, and most recently, COVID‐19, which has affected the world [16, 17, 18, 19]. In this article, we aim to use nomograms to build a prediction model for SFTS‐related encephalitis to provide front‐line clinicians with a simple and reliable tool, making it possible to identify and intervene early in potential encephalitis patients, thereby improving patient prognosis.

## 2 Materials and Methods

### 2.1 Study Subjects

We conducted a retrospective analysis of confirmed SFTS patients admitted to Nanjing Drum Tower Hospital in Nanjing, Jiangsu Province, China, between May 2019 and January 2024. The inclusion criteria were as follows: The diagnosis of SFTS was confirmed based on the following criteria: ①Positive SFTSV nucleic acid detection in clinical specimens. ②Clinical specimens showing a positive conversion or a more than fourfold increase in SFTSV‐specific IgG antibody levels during the recovery period compared to the acute phase. ③Isolation of SFTSV from clinical specimens. Diagnosis is possible if one of the above factors is present [20]. Criteria for clinical diagnosis of encephalopathy are defined as those meeting the following criteria: 1. Major criteria (required): The patient presents with an altered mental status (defined as decreased or altered level of consciousness, somnolence, or personality change) that lasts for ≥ 24 h and no other cause is identified. 2. Minor criteria: ① Fever ≥ 38° C (100.4 °F) recorded within 72 h before and after presentation; ② Generalized or partial seizures not entirely attributable to previous epilepsy; ③New positive neurological examination results; ④Cerebrospinal fluid white blood cell count ≥ 5/cubic millimeter; ⑤Neuroimaging showing abnormal brain parenchyma suggesting encephalitis, which was either new in previous studies or acute; ⑥Abnormal electroencephalogram and those related to the brain inflammation and other causes were ruled out [21]. Criteria for clinical diagnosis of SFTS‐related encephalopathy: patients who meet both the SFTS criteria and the major criteria for encephalopathy and two or more minor criteria [22]. Exclusion criteria: ① Underage patients (under 18 years); ② Patients with incomplete data.

Finally, we included a total of 220 patients with confirmed SFTS, of whom 47 were diagnosed with SFTS‐related encephalitis. The following sample size calculation formula was used to assess whether the sample size meets the requirements: *n* = Z2 × (P × (1‐P))/E2 (Z: At the 95% confidence level, *Z* is 1.96; E: Expected margin of error; *p* = 0.05). Then, the patients were randomly divided into training and validation sets. The training set included 154 patients (70% of the total), while the validation set consisted of 66 patients (30% of the total).

Our study was approved by the Ethics Committee of Nanjing Drum Tower Hospital. Because this study was a retrospective study, patient consent was not required. This study complies with the 1964 Declaration of Helsinki and its later amendments.

### 2.2 Data Collection

We collected patients' demographic information (gender, age, underlying diseases, etc.), relevant clinical manifestations, and first laboratory test results (including viral load, biochemical tests, coagulation function, etc. from the hospital's electronic medical record system), as well as comorbidities and prognosis. To reduce the bias caused by erroneous information during data entry, we arranged a second data entry operator to check the data. All data entry personnel received relevant training. The observation endpoint of this investigation was whether encephalitis developed (Table 1).

**Table 1:** General situation.

	Total	Non‐SFTSAE	SFTSAE	*P*
Number of patients	154	126	28	
**Demographics**				
Age, years, mean ± SD	62.56 ± 11.54	61.32 ± 11.55	68.14 ± 9.88	0.004
Female, *n* (%)	83 (53.9%)	69 (54.76%)	14 (50%)	0.648
Hospital stay (days)	10.20 ± 7.22	9.86 ± 5.33	11.75 ± 12.68	0.445
ICU treatment, *n* (%)	20 (12.99%)	10 (7.94%)	10 (35.71%)	< 0.001
**Underlying disease**				
Hypertension, *n* (%)	44 (28.57%)	32 (25.4%)	12 (42.86%)	0.064
Diabetes, *n* (%)	19 (12.34%)	15 (11.9%)	4 (14.29%)	0.752
**Clinical symptoms, *n* (%)**				
Fever, *n* (%)	152 (98.7%)	124 (98.41%)	28 (100%)	1.00
‐Max temp. (°C)	38.98 ± 0.62	38.96 ± 0.65	39.10 ± 0.49	0.294
‐Fever duration (Days)	9.18 ± 4.11	8.5 ± 3.47	12.25 ± 5.27	＜0.001
Gastrointestinal symptoms, *n* (%)	98 (63.64%)	78 (61.9%)	20 (71.43%)	0.343
Feeble, *n* (%)	135 (87.66%)	107 (84.92%)	28 (100%)	0.025
Hemorrhagic manifestations, *n* (%)	34 (22.08%)	18 (14.29%)	16 (57.14%)	＜0.001
**Death, *n* (%)**	27 (17.53%)	13 (10.32%)	14 (50%)	＜0.001

*Note:* Comparison of general baseline characteristics between encephalitis and non‐encephalitis groups.

### 2.3 Statistical Analysis

Statistical analyses were performed using SPSS version 27 (SPSS Inc., Chicago, IL, USA) and R (version 4.2.3). The Shapiro–Wilk test was used to test whether the data conformed to the normal distribution. For comparisons between data groups that conformed to a normal distribution, the independent sample *t*‐test was used. The results were expressed as mean ± standard deviation. Non‐normally distributed data were expressed as median and interquartile range, and comparisons between groups were performed using the Mann–Whitney U test. The Chi‐square test was used for categorical variables. For data that did not meet the chi‐square test conditions, Fisher's exact test was used and expressed as percentage (*n*, %). Single‐factor and multi‐factor logistic regression analyses were used to determine the independent risk factors for the occurrence of encephalitis. The selected independent risk factors were used to establish a model to evaluate the occurrence of encephalitis, and a nomogram was drawn based on this model. The area under the receiver operating characteristic curve (AUC) of the nomogram was calculated. Finally, the model was validated using the validation set to evaluate whether the model was practical.

## 3 Results

### 3.1 Demographics and Clinical Symptoms

Our study involved a total of 220 patients who met the inclusion criteria, among whom 154 patients were randomly selected as the training set to screen for independent risk factors of SFTS‐associated encephalitis and construct a nomogram. Our sample size met the calculated predicted values. The general characteristics of these 154 patients are shown in Table 1. Among them, 83 (53.9%) were female; the occurrence of SFTSAE did not show gender difference. The average age of all patients was 62.5 years, and the age of patients in the SFTSAE group was significantly higher than that in the non‐SFTSAE group, with statistical significance, indicating that age plays an important role in the progression of SFTSAE. There was no statistically significant difference in the length of hospital stay between the encephalitis group and the non‐encephalitis group, but the average hospital stay was longer and more variable in the encephalitis group. This may be because some encephalitis patients died soon after admission, while those who survived had a longer hospital stay. The probability of ICU admission was significantly higher in the encephalitis group (*p* < 0.001), suggesting that encephalitis poses a significant threat to patients. Upon admission, 57 patients (37.0%) in the encephalitis group had underlying conditions (defined as hypertension, coronary artery disease, or diabetes).

Regarding clinical symptoms, almost all patients had fever. Interestingly, the p‐value for the presence of fever between the encephalitis group and the non‐encephalitis group was 1, indicating that fever is widespread among SFTS patients. Meanwhile, the duration of fever was significantly longer in SFTSAE patients. A total of 135 (87.66%) patients experienced fatigue, and 98 (63.64%) patients had gastrointestinal symptoms (defined as diarrhea, abdominal pain, or nausea/vomiting). Thirty‐four (22.08%) patients had bleeding symptoms (defined as any of the following: eye bleeding, subcutaneous bleeding, gum bleeding, nosebleed, hematemesis, melena, hematuria, or bleeding at the puncture site), with significant differences between the encephalitis and non‐encephalitis groups. These suggest that SFTSAE patients are not only affected neurologically but also have considerable impacts on other systems, such as the coagulation and motor systems.

Finally, the mortality rate in the encephalitis group was significantly higher than in the non‐encephalitis group (*p* < 0.001), further indicating the severe adverse impact of encephalitis on SFTS patients. Researchers should conduct more studies targeting SFTSAE.

### 3.2 Laboratory Parameters

We compared relevant laboratory parameters between the SFTSAE group and the non‐SFTSAE group, and the results are shown in Table 2. In the SFTSAE group, aspartate aminotransferase (AST), total bile acids (TBA), blood urea nitrogen (BUN), serum creatinine (Scr), C‐reactive protein (CRP), creatine kinase (CK), creatine kinase‐MB (CK‐MB), Troponin T(Tnt), procalcitonin (PCT), d‐dimer, and viral load were higher. Platelet count (PLT), total cholesterol (TC), high‐density lipoprotein cholesterol (HDL‐C), and low‐density lipoprotein cholesterol (LDL‐C) were lower. Prothrombin time (PT), thrombin time (TT), and activated partial thromboplastin time (APTT) were prolonged. These indicators suggest that the damage of SFTS patients involves many aspects such as the heart, liver, coagulation, kidneys, etc. The functions of these organs are crucial for maintaining normal physiological functions of the human body. For other parameters, no statistically significant differences were observed between the two groups.

**Table 2:** Laboratory parameters.

Variables	Total (*n* = 154)	Non‐SFTSAE (*n* = 126)	SFTSAE (*n* = 28)	*P*
WBC (10^9/L)	3.1 (1.9,5.07)	2.85 (1.90,4.6)	4.45 (2.20,5.53)	0.115
Neu (10^9/L)	1.5 (1,3.3)	1.45 (1,2.8)	2.7 (1.32,4.25)	0.03
Lym (10^9/L)	0.8 (0.5,1.4)	0.8 (0.5,1.4)	0.7 (0.57,1.2)	0.949
Mono (10^9/L)	0.2 (0.1,0.4)	0.2 (0.1,0.38)	0.2 (0.1,0.4)	0.603
RBC (10^12/L)	4.31 (3.86,4.71)	4.3 (3.82,4.71)	4.32 (3.95,4.7)	0.933
Hb (g/L)	129.45 ± 18.69	129.58 ± 17.75	128.89 ± 22.82	0.861
PLT (10^9/L)	56.5 (36,75)	59.5 (37.5,80)	42 (24.25,67.75)	0.009
ALT (U/L)	71.2 (43.58,100)	68.55 (40.5,100.65)	78.25 (52.25,100)	0.247
AST (U/L)	128 (75,246.22)	118.5 (70.75,226.7)	218.8 (98,384.25)	0.023
ALP (U/L)	63.95 (54.2,89.72)	62.4 (54.05,88.35)	70.8 (57.7,93.28)	0.218
TBA (umol/L)	4.6 (2.73,10.12)	4.2 (2.6,8.12)	9.15 (4.05,19.82)	0.013
BUN (mmol/L)	4.4 (3.27,5.98)	4.14 (3.2,5.36)	5.46 (4.37,7.71)	0.003
Scr (umol/L)	62.15 (50,78.88)	59.65 (48,72)	79.75 (64.72,99.47)	< 0.001
TG (mmol/L)	1.96 (1.38,2.9)	1.94 (1.42,2.9)	2.09 (1.37,2.49)	0.853
TC (mmol/L)	3.3 ± 0.85	3.39 ± 0.87	2.93 ± 0.68	0.01
HDL‐C (mmol/L)	0.88 ± 0.35	0.93 ± 0.34	0.69 ± 0.3	0.001
LDL‐C (mmol/L)	1.33 (0.93,1.84)	1.46 (0.96,1.9)	1.02 (0.7,1.46)	0.005
PCT (ng/ml)	0.12 (0.07,0.31)	0.1 (0.07,0.22)	0.33 (0.15,0.56)	< 0.001
CRP (mg/L)	4.25 (2.7,10.62)	3.8 (2.5,8.25)	8.77 (4.9,45.15)	< 0.001
CK (U/L)	340.5 (115.5,717.5)	292 (105.75,690.75)	615 (257.75,1304.75)	0.009
CK‐MB (U/L)	19.5 (14,32)	18.5 (14,31)	25 (15,34.25)	0.171
Tnt (ug/L)	0.02 (0.01,0.06)	0.02 (0.01,0.05)	0.05 (0.02,0.12)	0.001
PT (sec.)	11.35 (10.5,12.3)	11.2 (10.4,12.1)	12.25 (11.28,12.9)	0.002
APTT (sec.)	36.3 (31.65,42.8)	35.75 (31.2,40.62)	43.85 (37.7,51.02)	< 0.001
TT (sec.)	22.05 (19.92,28.43)	21.4 (19.9,25.75)	31.6 (22.15,49.65)	< 0.001
Fib (g/L)	2.3 (2,2.7)	2.4 (2,2.7)	2.2 (1.87,2.52)	0.23
D‐D (mg/L)	2.08 (0.96,5.54)	1.86 (0.84,4.17)	5.08 (1.88,10.14)	0.002
Viral Load (lg copy/mL)	5 (4–6)	5 (4–6)	6 (5–7)	< 0.001

*Note:* Characteristics of the 154 patients with severe fever with thrombocytopenia syndrome enrolled in the training set.

Abbreviations: ALP, Alkaline Phosphatase; ALT, Alanine Aminotransferase; APTT, Activated Partial Thromboplastin Time; AST, Aspartate Aminotransferase; BUN, Blood Urea Nitrogen; CK, Creatine Kinase; CK‐MB, Creatine Kinase‐MB; CRP, C‐Reactive Protein; D.D, d‐Dimer; Fib, Fibrinogen; Hb, Hemoglobin; HDLC, High‐Density Lipoprotein Cholesterol; LDLC, Low‐Density Lipoprotein Cholesterol; Lym, Lymphocytes; Mono, Monocytes; Neu, Neutrophils; PCT, Procalcitonin; PLT, Platelets; PT, Prothrombin Time; RBC, Red Blood Cell; Scr, Serum Creatinine; TBA, Total Bile Acids; TC, Total Cholesterol; TG, Triglycerides; Tnt, Troponin; TT, Thrombin Time; WBC, White Blood Cell.

### 3.3 Selection of Predictive Factors

**Figure 1:**
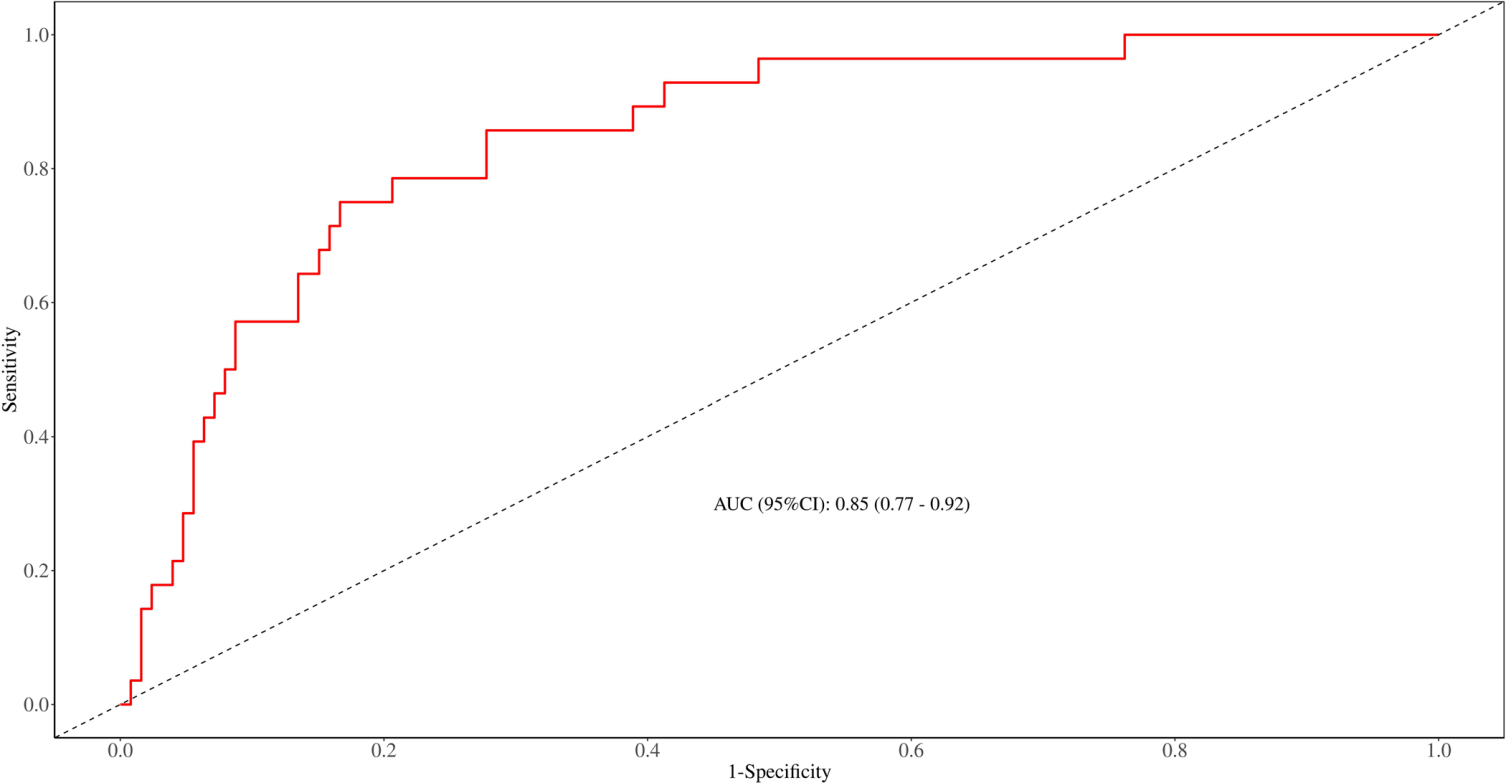
ROC curve of the training set. ROC curve and AUC value of the prediction nomogram constructed based on the 4 risk factors of this study. (Training set).

**Figure 2:**
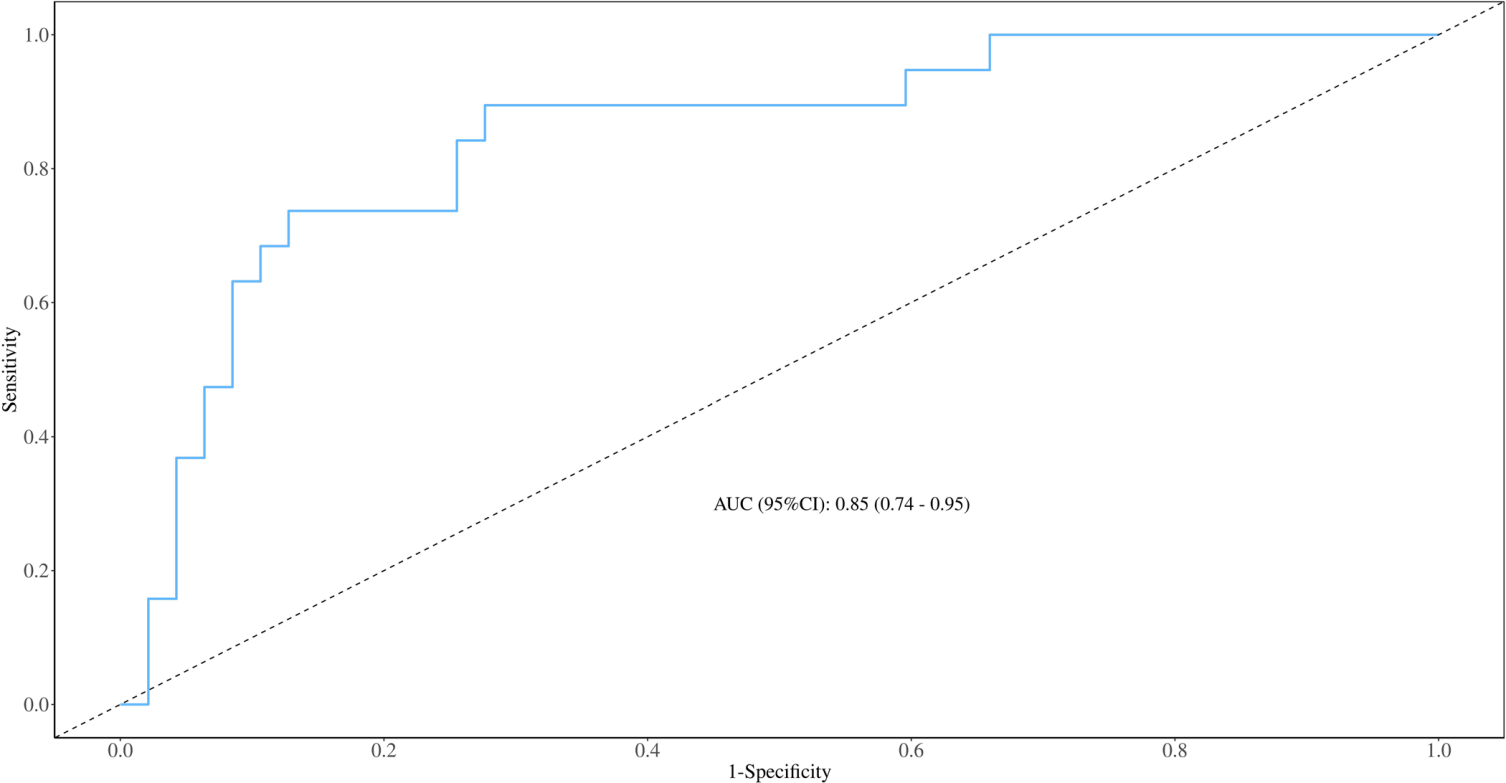
ROC curve of the validation set. The figure shows the ROC curve and AUC value of the validation set.

**Figure 3:**
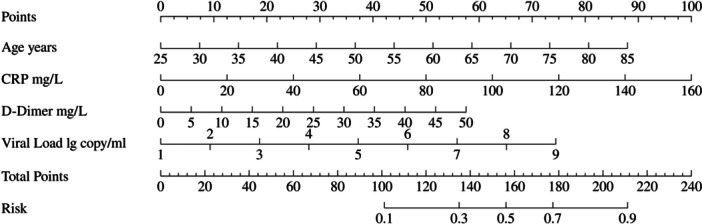
Nomogram. The predictive nomogram constructed based on age, CRP levels, d‐dimer levels, and Viral Load. Nomo‐score = (Intercept)*−8.076692 +Age* 0.058582 + CRp* 0.024975 + d‐dimer* 0.045992+ Viral Load * 0.371924.

The results of univariate logistic regression indicated that advanced age, decreased platelet count, decreased total cholesterol, decreased high‐density lipoprotein cholesterol, decreased low‐density lipoprotein cholesterol, elevated blood urea nitrogen, elevated serum creatinine, elevated procalcitonin, elevated C‐reactive protein, elevated d‐dimer, prolonged prothrombin time, prolonged thrombin time, and increased viral load were significantly associated with the occurrence of SFTS‐associated encephalitis Table 3 and Figures 1, 2, 3.

**Table 3:** Single factor logistic regression.

Variables	Coefficient	OR (95%CI)	*P*
Age(years)	0.07	1.07 (1.02–1.12)	0.006
PLT (10^9/L)	−0.02	0.98 (0.96–0.99)	0.009
BUN (mmol/L)	0.12	1.13 (1.01–1.26)	0.043
Scr (umol/L)	0.01	1.01 (1.01–1.02)	0.025
TC (mmol/L)	−0.69	0.50 (0.29–0.86)	0.012
HDL‐C (mmol/L)	−2.21	0.11 (0.03–0.44)	0.002
LDL‐C (mmol/L)	−1.01	0.36 (0.18–0.75)	0.006
CRP (mg/L)	0.03	1.03 (1.01–1.05)	< 0.001
PCT (ng/ml)	0.75	2.11 (1.14–3.93)	0.018
PT (sec.)	0.39	1.48 (1.13–1.94)	0.005
TT (sec.)	0.02	1.02 (1.01–1.03)	0.011
d‐Dimer (mg/L)	0.05	1.05 (1.01–1.09)	0.013
Viral Load (lg copy/ml)	0.53	1.70 (1.25–2.32)	< 0.001

*Note:* Single factor logistic regression results. These indicators involve multiple aspects, indicating that SFTS can affect multiple organs.

To further identify predictors of SFTSAE in patients, multivariate logistic regression analysis was performed on these indicators. The results showed that advanced age, elevated C‐reactive protein, elevated d‐dimer, and high viral load were independent risk factors for the development of encephalitis in SFTS patients. According to the final number of independent risk factors, we believe that the data size included in this study is appropriate. A predictive nomogram was constructed based on these four indicators, and the AUC was calculated. The nomogram result is Nomo‐score = (Intercept)*−8.076692 +Age* 0.058582 + CRp* 0.024975 + d‐dimer* 0.045992+ Viral Load* 0.371924. The AUC was 0.846, 95% confidence interval [CI] of 0.770–0.921. The AUC for the validation set was 0.848, 95% CI of 0.744–0.952. These results indicate that the model can reasonably predict the likelihood of encephalitis in patients, aiding in the early identification of potential encephalitis cases Table 4.

**Table 4:** Multifactor logistic regression.

Variables	Coefficient	OR (95%CI)	*P*
Age (years)	0.06	1.06 (1.01‐1.12)	0.027
CRP (mg/L)	0.02	1.03 (1.01‐1.04)	0.007
d‐Dimer (mg/L)	0.05	1.05 (1.01‐1.09)	0.042
Viral load (lg copy/ml)	0.37	1.45 (1.03‐2.05)	0.035

*Note:* Multifactor logistic regression results. These indicators reflect the factors most likely to influence the occurrence of encephalitis in SFTS patients.

## 4 Discussion

Severe Fever with Thrombocytopenia Syndrome, a recently identified infectious disease, is characterized by a relatively high overall case fatality rate. According to existing reports, the case fatality rate ranges from 16.2% to 30%, aligning with our sample rate of 17.53% [1, 5]. Previous studies have reported neurological complications in SFTS patients, prompting concerns regarding these complications [15, 23]. The incidence of SFTS‐associated encephalitis is reported to be between 19.1% and 22.7%, which closely matches our sample rate of 18.2% [14, 15]. However, the case fatality rate for SFTS‐associated encephalitis in our sample was 50%, slightly higher than previously reported figures. To improve the prognosis of SFTS patients through early detection of encephalitis, we reviewed relevant data from SFTS patients, including demographic information, clinical presentations, initial laboratory findings upon admission, complications, and outcomes. We divided the patients into training and validation cohorts to develop a model capable of early prediction of SFTS‐associated encephalitis, which demonstrated good predictive performance. Statistical analysis revealed that advanced age, elevated CRP levels, increased d‐dimer levels, and high viral load were significantly associated with the occurrence of SFTS‐associated encephalitis.

Advanced age has been widely recognized as a risk factor for poor prognosis in SFTS [24, 25], and is even considered one of the most significant risk factors [26]. This finding is also supported by animal models, where, for example, in a ferret‐based model, researchers observed that the virus replicates more readily in older ferrets [27]. The fact that the inflammatory storm has a fatal impact on SFTS patients has been widely reported, and elderly patients typically experience more severe inflammatory storms [28, 29]. This also involves another indicator, C‐reactive protein. C‐reactive protein usually represents the severity of inflammation. This shows that encephalitis usually occurs in patients with severe inflammatory response, which is consistent with general knowledge. Systemic inflammation can increase blood–brain barrier permeability and increase the risk of central nervous system infection, which is a classic pathophysiological process observed in many studies of viral encephalitis [30]. In previous studies on SFTSAE, researchers found significantly elevated levels of inflammatory markers (MCP‐1 and IL‐8) in the cerebrospinal fluid of SFTSAE patients, further highlighting the crucial role of inflammation in the development of SFTSAE [23].

Coagulopathy is a common clinical manifestation in SFTS patients. SFTS virus can replicate within vascular endothelial cells [31] and directly cause vascular damage. This will manifest as an increase in d‐dimer levels. Inflammation resulting from vascular injury further increases vascular permeability, allowing SFTSV to enter the central nervous system through the compromised blood–brain barrier [32].

SFTSV viral load also plays a crucial role in the development of SFTSAE [33, 34]. As mentioned above, SFTSV itself can directly attack blood vessels or the central nervous system and cause severe inflammation. The high viral load has many viruses, which will lead to more obvious inflammation and central nervous system damage, resulting in severe clinical symptoms. Therefore, we recommend that clinicians closely monitor these indicators to reduce the likelihood of SFTSAE and improve clinical outcomes.

This study investigated the independent risk factors for SFTS‐associated encephalitis, including age, d‐dimer levels, elevated C‐reactive protein levels, and high viral load. Based on these four factors, we developed a nomogram to assess the likelihood of encephalitis in SFTS patients. To our knowledge, this is the first clinical predictive model based on multivariate logistic regression for predicting SFTS‐associated encephalitis. We believe that this simple and effective model will assist clinicians in making critical decisions. The development of predictive models can help clinicians identify potential SFTSAE patients earlier, allowing for targeted treatment to improve patient outcomes.

There are several limitations to this study. As a single‐center retrospective study, the quality and generalizability of the data may be affected. Additionally, our model has not yet undergone external validation, and future prospective studies will be conducted to evaluate the model's effectiveness. The above limitations may reduce the external validity of the model.

## 5 Conclusion

In conclusion, our study identified several critical risk factors for patients with SFTSAE, including older age, elevated C‐reactive protein levels, elevated d‐dimer levels, and high viral load. A risk nomogram was developed based on these indicators for early assessment of the risk of SFTS patients developing encephalitis, which may play a significant role in guiding future clinical treatment.

## Author Contributions

**Yijiang Liu:** conceptualization, methodology, writing–original draft. **Naisheng Zhu:** formal analysis. **Zimeng Qin:** data curation. **Chenzhe He:** data curation. **Jiaqi Li:** data curation. **Hongbo Zhang:** data curation. **Ke Cao:** conceptualization, methodology, writing–review and editing. **Wenkui Yu:** conceptualization, methodology.

## Ethics Statement

Our study protocol was approved by the human research ethics committee of Nanjing Drum Tower Hospital, with the ethical approval number 2023‐061‐01. This study is performed in accordance with the Helsinki Declaration of 1964 and its later amendments.

## Consent

Patient consent is waived by the ethics committees because of the retrospective nature of the study. All named authors meet the International Committee of Medical Journal Editors (ICMJE) criteria for authorship for this article, take responsibility for the integrity of the work as a whole, and have given their approval for this version to be published.

## Conflicts of Interest

The authors declare no conflicts of interest.

## Supporting information

Supporting information.

## Data Availability

The data that support the findings of this study are available from the corresponding author upon reasonable request.
